# Studying the Experiences of Children With Moderate to Profound Intellectual Disabilities in Research: A Systematic Review

**DOI:** 10.1111/jir.70009

**Published:** 2025-07-12

**Authors:** Satu Peltomäki, Sirpa Granö, Ella Rönkkö, Raija Pirttimaa, Kirsi Pyhältö, Elina Kontu

**Affiliations:** ^1^ University of Helsinki Helsinki Finland; ^2^ Tampere University Tampere Finland; ^3^ University of Jyväskylä Jyväskylä Finland

**Keywords:** adaptation, adolescent, data collection, intellectual disability, participation, systematic review

## Abstract

**Background:**

Children with moderate to profound intellectual disabilities are not usually invited to share their lived experiences through research. In addition to their difficulties in communication and cognitive functions, their exclusion is caused by the lack of suitable data collection instruments. Since the 1990s, more research has focused on the lived experiences of people with intellectual disabilities. The aim of this review is to examine how the experiences of children with moderate to profound intellectual disabilities have been studied.

**Method:**

A systematic review was conducted in accordance with the PRISMA statement. Studies were searched from CINAHL, Education Collection, Sociology Collection, Social Science Database and PsycINFO in November 2023 and January 2025. Eligible studies had been published since the year 2000 in English or Finnish, included at least one child with moderate to profound intellectual disabilities and aimed to collect their experiences. General study details, population characteristics, and data collection details were extracted. The methodological quality was assessed with the Mixed Methods Appraisal Tool (MMAT). The analysis included a descriptive summary and inductive content analysis with intercoder reliability assessment.

**Results:**

The eligible studies (*n* = 41) used individual interviews, questionnaires, task‐based approaches, observations and focus groups. Participants were mainly 12 years or older, had moderate intellectual disabilities, and could communicate with speech. The adaptations (i.e., any adjustments made for specific needs) comprised four top‐level categories and 27 subcategories. The most commonly used adaptations and adaptations for different participant groups are presented.

**Conclusions:**

Individual interviews and questionnaires with simple adaptations usually suit children with moderate intellectual disabilities who use speech to communicate. Individual interviews with multiple adaptations can be suitable for a wider group. Some task‐based approaches and profound and long‐lasting observations can be considered with children with non‐symbolic communication and severe or profound intellectual disabilities.

## 1 Introduction

People with intellectual disabilities have the same rights as anyone else to share their lived experiences (United Nations 1991, 2006). Furthermore, sharing one's lived experiences is a key factor in making life the way one wants it to be, and being understood by others (Shier 2006). Sharing lived experiences through research enables having an impact on society, in addition to the direct benefits of the studies on their participants. Sharing lived experiences as a child enhances the skills to share them later. Research on the lived experiences of people with intellectual disabilities has increased since the 1990s. For example, a search in Education Collection (ProQuest) shows that the terms ‘participatory’ and ‘intellectual disability’ have been used in studies since the early 1990s, while the terms ‘medical’ and ‘intellectual disability’ have been used since the 1930s. The initial impetus for research to focus on the perspectives of people with intellectual disabilities include the growth of the disability movement with James Charlton's phrase ‘Nothing about us without us’ in 1993, which implies that people with disabilities should be part of anything that concerns them, in addition to the Salamanca statement in 1994 where the principle of inclusion was presented. The discussion has moved from the medical toward the social model of disability, in which society is seen as causing the disabling of people and causing their exclusion, rather than the individuals' impairments (Oliver 2004; Shakespeare and Watson 1997).

Despite the societal change and growth in research since the 1990s, researchers still face several methodological challenges when including people with intellectual disabilities as informants, especially as the participants' disabilities become more complex (Maes et al. 2020). These challenges lie in data collection and analysis, ethics, participant demarcation (e.g., different terminologies between countries, heterogeneity of the group) and recruitment, and applicability of theoretical models (Maes et al. 2020; Mietola et al. 2017). Participating in traditional data collection requires skills such as understanding abstract topics, contributing ideas and topics for discussion, working independently, answering according to one's own wishes and remembering and reflecting on past events. These skills are often difficult for people with ID, including children with moderate to profound intellectual disabilities (Gjermestad et al. 2017). Earlier reviews have shown that people with intellectual disabilities who need support in communication and cannot communicate ‘well enough’ are usually excluded from being research informants (Bailey et al. 2014; Corby et al. 2015; DePape and Lindsay 2015; Eisen et al. 2018; Tesfaye et al. 2019). Instead, data are collected from proxies who present their interpretations of the person's lived experiences or behaviour observation, which focuses on functioning rather than experiences (Maes et al. 2020). Proxy reports can provide important and unique information (Kruithof et al. 2024; Nieuwenhuijse et al. 2024). However, studies have also reported disagreements between proxies and people with intellectual disabilities (Santoro et al. 2022). Eventually, proxies' personal beliefs, hopes and goals cannot be separated from their interpretative answers (see also Maes et al. 2020).

Children with moderate to profound intellectual disabilities are a diverse group. The description of intellectual disability by the American Association on Intellectual and Developmental Disabilities (AAIDD) includes ‘significant limitations in both intellectual functioning and adaptive behaviour as expressed in conceptual, social, and practical skills’ (Schalock et al. 2021). The World Health Organization has specified that most children and adolescents with mild intellectual disabilities can communicate effectively, while the support needs in communication and other skills expand with children and adolescents with moderate, severe and profound intellectual disabilities (WHO 2024). These classifications provide some basic assumptions about children with moderate to profound intellectual disabilities, the target group of the current review; they need support to take part as research informants. However, diagnostics are always indicative, especially in borderline cases. At one end of the target group are children with profound intellectual and multiple disabilities (PIMD) with an ‘idiosyncratic communicative repertoire’ who communicate non‐symbolically with facial expressions, movements, vocalisations and other subtle signals (Nakken and Vlaskamp 2007). Many children use symbolic augmentative and alternative communication (AAC) methods, such as pictures and key word signs (Huuhtanen 2012). At the other end of the spectrum are children with borderline to moderate intellectual disabilities who can read and write and understand many abstract concepts. Each child on the spectrum has individual abilities and needs. What is important is to provide each person with the support they need, despite their diagnosis (Schalock et al. 2021). Thus, with support and attentiveness of the communication partner, and the right methods, many children in this group can share their likes, dislikes and needs, with these sometimes being a pointer to a current feeling or an immediate preference communicated with facial expressions and physical gestures (Kruithof et al. 2024; Mietola et al. 2017; Miettinen 2021; Skarsaune et al. 2021; Ware 2004). This requires research methods to be developed or adapted specifically for pursuing their lived experiences.

In the current review, we discuss studying experiences. Life is experienced through consciousness, after which these experiences can be remembered and described, but memories are always incomplete subjective reconstructions (Dewey 1920). We appreciate that the emotions, narratives, opinions and other first‐person points of view are valuable ways of understanding and describing experiences. These include immediate preferences and non‐symbolic communication that, on their own, are incomplete ways of describing a person's experience, but in many cases, they are the only pointers that can be reached from the outside and thus are worth striving for (Miettinen 2021; Skarsaune et al. 2021; Ware 2004).

The aim of this systematic review is to examine how the experiences of children with moderate to profound intellectual disabilities have been studied. The group is diverse, and eventually, their support needs must be evaluated individually. A diverse group was chosen for this review to get results that could be applied to a wider group. Some instruments and adaptations are presumed to be suitable for many children with moderate ID, and some are perhaps more suitable for children with severe or profound ID. To be able to find proper individual support methods, it is important to understand the variations they consist of. This is the gap that the current review aims to fill. Our review questions are:

1. What data collection instruments have been used to collect data about the experiences of children with moderate to profound ID?
2. What adaptations have been used with these instruments?

## 2 Methods

### 2.1 Search Strategy

The current review was conducted under the Preferred Reporting Items for Systematic Reviews and Meta‐Analysis (PRISMA) statement (Page et al. 2021) in CINAHL, Education Collection, Sociology Collection, Social Science Database and PsycINFO. The search, conducted by the first author, included terms that represented the sample (intellect*/developmental/learning AND disab* AND moderate*/severe*/profound*) and evaluation (experience*/view/participatory*/inclusive*/own voice*/authentic voice*/giving voice*) (Appendix A Tables A1–A3) (Cooke et al. 2012). The terms that represented the sample aimed to target a variety of countries (Maes et al. 2020). A research librarian at the University of Helsinki was consulted regarding the databases and search terms. The databases focus on education, psychiatry, the social sciences and social work, which represent the principal services required by our target group. The systematic search was conducted on 7 November 2023 and repeated on 29 January 2025.

### 2.2 Eligibility Criteria and Study Selection

The inclusion criteria were as follows: the article (1) has been published in a peer‐reviewed journal in English or Finnish since the year 2000, (2) reported an empirical study, (3) included at least one participant with moderate to profound intellectual disabilities who was aged under 18 and (4) aimed to collect their experiences. We chose the year 2000 as the starting point because of the significant increase in relevant research from then. We noticed this in our preliminary searches, presumably due to the global emergence of an inclusive turn (e.g., United Nations 1991, 2006). We excluded studies that had neither stated that a participant had moderate to profound ID nor comprehensively described their representative skill levels. Experiences were seen as first‐person perspectives and narratives of previous, current and future events and self‐reported views and opinions of any subject matter. These can be communicated with any mode of communication, such as speech, writing, symbols, drawings and non‐symbolic communication.

**FIGURE 1:**
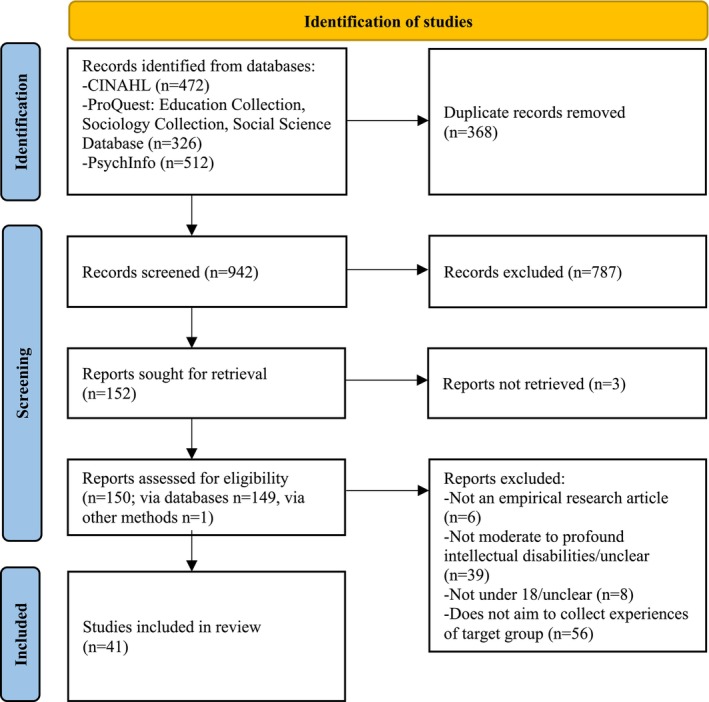
PRISMA flow diagram for the identification, screening and inclusion of studies.

We identified 1310 potentially relevant studies with the database searches (Figure 1). After removing 368 duplicates with Zotero (n.d.), 942 titles and abstracts were screened by the first author. We included all borderline records (i.e., those for which it was not certain if all inclusion criteria had been fulfilled). We retrieved 150 articles for full‐text examination. Two reviewers (authors SP and SG/RP/EK) assessed the eligibility of each full text. A kappa value of 0.84 showed a strong level of agreement. A discussion followed to resolve disagreements. The study selection resulted in 41 eligible studies (Table 1).

**TABLE 1:** Eligible studies.

No.	Authors, year	Data collection instruments for the target group's experiences	Experiences of …	Participant characteristics^a^						
				Mod. ID^b^	Sev. ID	Pro. ID	Speech	(Non‐) symbolic com.	≥ 12 Years	< 12 Years
1	Aderemi & Pillay, 2013	Questionnaire	Sexual abstinence and HIV	X			X		X	
2	Allen et al., 2022	Task‐based approach	Physical education lessons		X		X	X	X	X
3	Aviram et al., 2021	Focus group	Physical activity and exercise	X	X		X		X	
4	Benini et al., 2004	Task‐based approach	Pain	X			X		X	X
5	Bennett et al., 2017	Individual interview	Social relationships, independent work, school life and community inclusion	X	X	X	X	X	X	
6	Boyden et al., 2012	Individual interview	A mental health service	X			X		X	X
7	Byrne & Hennessy, 2009	Individual interview, questionnaire	Causes of challenging behaviour presented in a vignette and behavioural intentions toward peers	X			X		X	X
8	Caton & Kagan, 2007	Individual interview	Aspirations after leaving school	X			X		X	
9	Connaughton & Cline, 2020	Individual interview, observation	The good things in life	X			X		X	
10	Cooney et al., 2006	Questionnaire	Social comparisons, awareness of stigmatised treatment and future aspirations	X			X		X	
11	Dubé et al., 2023	Questionnaire	Anxiety, school climate and victimisation	X			X		X	X
12	Dubé et al., 2022a	Questionnaire	Relationship quality and depression	X			X		X	X
13	Dubé et al., 2022b	Questionnaire	Relationships with teachers, parents and peers, loneliness, school belonging, victimisation, self‐esteem and social behaviours	X			X		X	X
14	Fairhurst et al., 2018	Questionnaire	Pain and quality of life	X	X	X	X	X	X	
15	Ferreira et al., 2016	Task‐based approach	Friendship		X		X	X		X
16	Ferreira et al., 2019	Task‐based approach	Friendship	X	X		X	X		X
17	Fitzgerald, 2007	Task‐based approach	Physical education, sport, and free‐time activities		X		X	X		
18	Fitzgerald et al. 2003	Task‐based approach	Physical education and sporting		X		X	X	X	
19	Foley et al., 2012	Focus group	Wellbeing	X			X		X	X
20	Hatton et al., 2017	Individual interview	Health and bullying	X			X		X	
21	Hingley‐Jones, 2009	Observation	Life and emotions		X			X	X	
22	Hingley‐Jones, 2011	Observation	Life and emotions		X			X	X	
23	Hingley‐Jones, 2012	Observation	Life and emotions		X			X	X	
24	Hughes et al., 2013	Individual interview	Individual education plan involvement, self‐determination strategies and future goals		X		X		X	
25	Jenkin et al., 2017	Task‐based approach	Human rights needs and priorities		X		X	X	X	X
26	Kumas et al., 2024	Questionnaire	An early arithmetic skills training programme	X			X			X
27	Larkin et al., 2011	Individual interview	Contextual features of conflict	X			X		X	
28	Medved & Brockmeier, 2004	Individual interview	Major life events, family and peer roles	X			X		X	
29	Noerr & Swinford, 2024	Questionnaire	Adherence in a physical activity intervention	X			X		X	
30	Pearlman & Michaels, 2019	Individual interview	Life at school and home, and future aspirations	X	X	X		X	X	X
31	Pijl & Frostad, 2010	Task‐based approach, questionnaire	Friends and self‐concept	X	X		X		X	
32	Retznik et al., 2021	Individual interview	Partnership, sexuality, and contraception	X			X		X	
33	Ring & Travers, 2005	Individual interview	Educational provision		X		X			
34	Robertson et al., 2018	Individual interview	Sport, exercise, bullying and friendships	X			X		X	
35	Samuelsson et al., 2023	Individual interview	Speaking and reading	X	X		X	X	X	X
36	Valiquette et al., 2010	Individual interview	Communication, satisfaction and priorities	X			X	X	X	X
37	Vogel & Reiter, 2003	Individual interview	Bar/bat mitzvah ceremony	X			X		X	
38	Warnick et al., 2024	Questionnaire	Mental health	X			X		X	
39	Whitehurst, 2006	Individual interview	Drama production		X		X	X	X	X
40	Young et al., 2015	Individual interview, questionnaire	Worries and anxiety	X			X		X	
41	Young‐Southward et al., 2017	Individual interview	Transition from school to adulthood	X	X		X		X	

^a^ The participant characteristics are indicative.

^b^ ID: intellectual disability.

### 2.3 Data Extraction and Methodological Quality Appraisal

Our data extraction tool was used by the first author in Atlas.ti 23 software by creating a Report for each eligible study. The tool included (a) general study details (authors, countries, publication year, aim, type of experiences and central results), (b) population characteristics (age, cognitive and communication skills) and (c) data collection (descriptions of the instruments used to collect data of the target group's experiences, including the adaptations, i.e., any adjustments made for specific needs and other data collection).

Two reviewers (authors SP and SG/ER/RP/EK) independently appraised the quality of the eligible studies using the Mixed Methods Appraisal Tool (MMAT) (Hong et al. 2018) (Appendix B). The tool consists of five study design categories and five design‐specific questions. Discrepancies were resolved through mutual discussions. All studies were included in the results, Sections 3.1 and 3.2.1 to get a comprehensive review of the research field. Studies with at least three ‘yes’ answers in the MMAT evaluation were included in Sections 3.2.2 and 3.2.3 to present studies with good methodological quality when presenting the adaptation options for more specific participant groups.

### 2.4 Data Analysis

**FIGURE 2:**
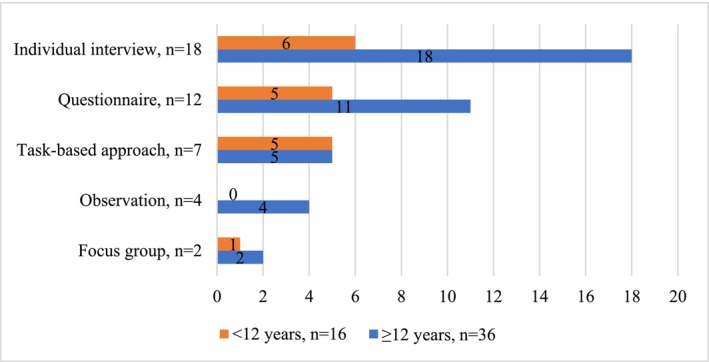
Participants' ages.

**FIGURE 3:**
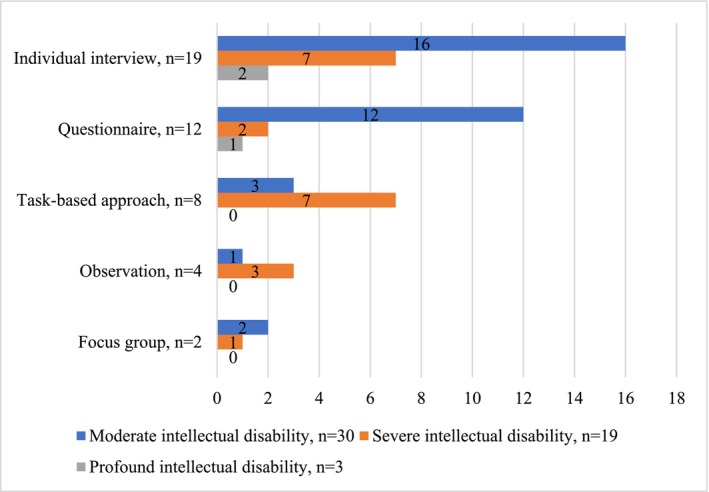
Participants' levels of intellectual disability.

**FIGURE 4:**
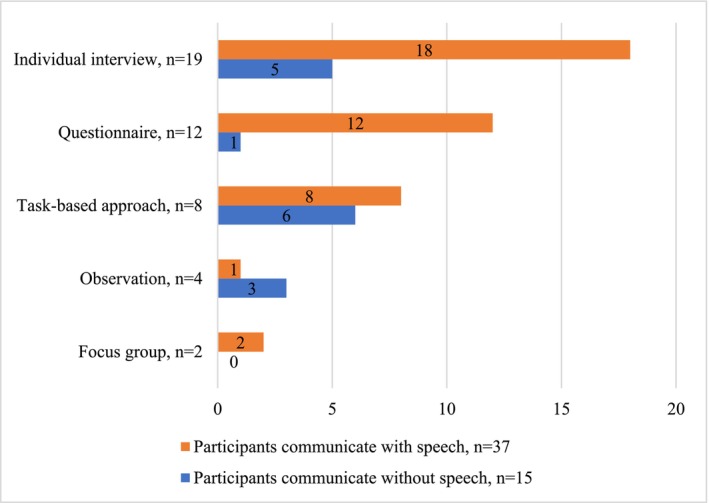
Participants' communication skills.

Data were analysed by the first author using Atlas.ti 23 software. Any uncertainties were resolved through mutual discussions. A descriptive summary (Cohen et al. 2018, 727) was used to answer our first review question. This comprised identifying the data collection instruments that studied the target group's experiences and participant characteristics (age, intellectual disability level and communication skills) and forming Figures 2, 3, 4. Communication skills were divided into communicating with speech and communicating without speech, the latter including symbolic communication (e.g., pictures, signing, single words and syllables) and/or non‐symbolic communication (e.g., facial expressions, movements, vocalisation and other subtle signals). The data extracted were summarised according to the instruments' descriptions.

Content analysis was used to answer our second review question and comprised five stages:

1. Inductive coding. Conceptually meaningful data segments were formed (O'Connor and Joffe 2020). Each segment illustrated one or multiple adaptations. The segments were labelled with codes that each summarised an adaptation described in the study.
2. Reading codes and forming categories. Sub‐categories (*n* = 27) and top‐level categories (*n* = 4) were identified from the codes (e.g., code; Additional drawings were used if needed, sub‐category; Drawing/writing, top‐level category; Communication support).
3. Verification of the previous stages. Data segments were divided when necessary, so each segment included only one sub‐category.
4. Intercoder reliability assessment. The process was undertaken by two coders (authors SG and EK) in six studies that together formed a representative sample of all sub‐categories (O'Connor and Joffe 2020). The coders inserted the sub‐categories into the data segments. Krippendorff's Alpha (among authors SP, SG and EK) ranged between 0.809 (top‐level category ‘methodological principles’) and 0.972 (top‐level category ‘guidance’).
5. Narrative exploring. Relationships within and across the eligible studies were explored by comparing Tables 1 and 2, in addition to the codes, sub‐categories, top‐level categories and extracted data. We used Atlas.ti code co‐occurrence analysis to identify the proportion of studies that used each adaptation with different participant characteristics groups. The results of the MMAT checklist were acknowledged by emphasising high‐quality studies (studies with at least three answers in ‘yes’) in the results when possible.

**TABLE 2:** Reported adaptations across the data collection instruments.

	Methodological principles	Communication support	Guidance	Collaboration
Individual interview	Experiences before testing [27;35;40] From knowing to knowledge [27;28;32] From one instrument to another [24;32] Time is flexible [6;35;40]	Auditive [30] Drawing/writing [6;33] Non‐verbal cues [5;39] Objects [30;39] Pictures/symbols/signing [6;28;30;32;35; 36;39;40] Plain language [6;32;36]	Asking examples [24] Emotional support [7;28;35;36;39] Practising [35;40] Repetition [5;35;36] Rephrasing [6;24;39]	Consultation (professionals) [35;39] Data collector (prof.) [30;39] Support people (family/friends) [36;41] Support people (prof.) [5;6;30;35]
Questionnaire	Experiences before testing [10] Functionality [10]	Pictures/symbols/signing [1;10–13] Plain language [1]	Asking examples [10] Cutting‐off questions [7] Emotional support [10–13;31] Rephrasing [1;7;10–13;31] Reading aloud [10–13;26;29;31]	Consultation (prof.) [7] Support people (family/friends) [29] Support people (prof.) [1;38]
Task‐based approach	From knowing to knowledge [2;17;18;25] Functionality [2;17;18;25] Time is flexible [2]	Auditive [25] Drawing/writing [2;18;25] Non‐verbal cues [17;18;25] Objects [4;18;25] Pictures/symbols/signing [2;4;15–18;25]	Emotional support [2;17;18;25] Peer support [17;18] Practising [4,15] Repetition [17;18] Rephrasing [2;4;18;25]	Consultation (co‐researcher) [2] Consultation (family/friends) [25] Consultation (prof.) [2;4;17;18;25] Data collector (co‐researchers) [25] Data collector (prof.) [18] Support people (prof.) [2;17;18]
Observation	From knowing to knowledge [22] Time is flexible [21;22]	Non‐verbal cues [21]		Consultation (family/friends) [21–23]
Focus group		Drawing/writing [19] Non‐verbal cues [19]	Emotional support [19] Providing examples [3] Rephrasing [19]	Data collector (prof.) [19] Support people (prof.) [19]

## 3 Results

### 3.1 Data Collection Instruments

Five main data collection instruments were identified from the eligible studies: individual interviews (*n* = 19), questionnaires (*n* = 12), task‐based approaches (*n* = 8), observations (*n* = 4) and focus groups (*n* = 2) (Table 1, Figures 2, 3, 4). As shown in Table 1, most studies (*n* = 37) used one of these instruments, with four studies using two instruments. In addition to using at least one of the instruments to study experiences, the studies collected data with proxy methods, behaviour observation and other methods that did not study the participants' experiences. These were not the focus of this review and were not part of the analysis or results. The studies' primary aim was to study either the target group's experiences or something else, such as the development of a specific skill in an intervention, and participants' experiences of the intervention formed part of the data.

Individual interviews were conducted mainly through a qualitative semi‐structured approach [6–8;27;28;32;33;40;41], numbering according to Table 1 or a quantitative structured approach [20;24;30;34–36]. Quantitative questionnaires included forced choices [7;29;40], Likert scales with numbers or words [1;7;10–14;29], ‘yes/no’ response formats [10;38] and open‐ended questions [26]. We used Fitzgerald et al. (2003) definition to define the task‐based approach, which is ‘[…] a process of engagement with learners in which they attempt to complete a series of tasks that require their active engagement rather than passive encounter’. Task‐based approaches were performed individually with qualitative and quantitative designs [2;4;15;16;25;31] and in groups with qualitative designs [17;18]. Four qualitative task‐based approaches were developed specifically for the target group [2;17;18;25]. Three studies used sociometric techniques and did not require the same active engagement as other task‐based approaches [15;16;31]. All studies with observation and focus groups had qualitative designs. Among the studies with observation, three were based on psychoanalytically informed infant observation, and were long‐lasting and in‐depth observations that were able to move past observing behaviour [21–23]. The method had similarities with ethnography, with the researchers immersing themselves into the families' lives, and the data comprising extensive field notes. The fourth study with observation had a traditional setting in which the interview and observation data supported each other. The informal, unstructured observations recorded discussions that the participant might not be able to provide in an interview [9]. The focus groups had at least two facilitators [3;19].

All five instruments were used with participants aged 12 years or older (Figure 2) with moderate and severe intellectual disabilities (Figure 3), and with participants who communicated with speech (Figure 4). Six studies (individual interviews, questionnaires and task‐based approaches) reported having excluded possible participants due to their communication difficulties or difficulties in self‐reporting [7;14–16;35;41]. The experiences of children with moderate intellectual disabilities who communicated with speech were studied mainly with individual interviews (*n* = 15) and questionnaires (*n* = 12). These studies reported challenges in not receiving long enough or nuanced answers from individual interviews [6;32;35;40], participants' difficulties in understanding the questionnaire's questions or scales [11–13;29], recall bias [1], acquiescence [7], and issues related to the adequacy of the questionnaire [10;38].

Children with severe intellectual disabilities who communicated with speech participated mainly in task‐based approaches (*n* = 7) and individual interviews (*n* = 6) (Table 1). These studies reported participants having difficulties in doing or understanding tasks [2;15;16;18;25] and understanding questions [35;39], reactivity [39] and the interview not providing sufficiently nuanced answers [35]. Children with severe intellectual disabilities who communicated (non‐)symbolically participated mainly in task‐based approaches (*n* = 6), individual interviews (*n* = 4) and observations (*n* = 3) (Table 1). Six of these studies included children with non‐symbolic communication modes and utilised profound and long‐lasting observations [21–23] and task‐based approaches with multiple adaptations [2;17;25]. Studies that included children with severe intellectual disabilities and (non‐)symbolic communication modes reported similar challenges as the studies that included children with severe intellectual disabilities who communicated with speech, in addition to difficulties in understanding participants' communication during tasks [17]. Three studies included children with profound intellectual disabilities and collected data with individual interviews [5;30] and a questionnaire [14]. The study collecting data with a questionnaire reported severe inclusion difficulties for non‐symbolic participants [14].

### 3.2 Reported Adaptations

In the current review, we defined adaptations as any adjustments made for the specific needs of the participants. At least one adaptation was reported in 35 studies. Six studies did not report adaptations [8;9;14;20;34;37]. These studies included children with moderate intellectual disabilities who communicated with speech, except for Study 14, which reported the inclusion of children with moderate to profound intellectual disabilities, in addition to many participants not being able to answer their questionnaire. We divided the adaptations into four top‐level categories (methodological principles, communication support, guidance, and collaboration) and 27 sub‐categories (Table 2; Appendix C). The methodological principles illustrated adaptations that were connected to the complete structure of the study. Communication support illustrated AAC methods, while guidance provided details of adapting speech‐based instructions. Collaboration included having different people supporting the research process. Table 2 presents the sub‐categories and their representative data collection instruments. Individual interviews [6;35] and a task‐based approach [2;17;18;25] reported adaptations from all top‐level categories, while a limited number of adaptations were reported in questionnaires, observations, focus groups and quantitative studies.

#### 3.2.1 The Most Used Adaptations

The most used adaptations were ‘pictures/symbols/signing’, ‘rephrasing’, and ‘emotional support’. In addition to a range of pictures, graphic symbols, and emotional faces, the sub‐category ‘pictures/symbols/signing’ included the use of tangible decision‐making [2;15;16;18;36;40] and Talking Mats (Murphy 1998) [35;39], Makaton (language programme) (Grove and Walker 1990) [2;17;18;30;39], photographs [6;15;16;25;28;39], interview tool designed for speech‐generating devices [36], and key word signing (Huuhtanen 2012) [17;25;35]. These AAC methods were used in individual interviews, questionnaires and task‐based approaches (Table 2). Studies usually combined various sub‐categories of communication [e.g., 18;39]. Supplementing questions with quality communication adaptations was important, especially when the researcher had not met the participants before and had little knowledge of their communication skills [6]. Most studies provided the same communication adaptations to all participants [1;4;10–13;15;16;32;35;36]. Some studies emphasised an individual approach in which communication support was planned individually, for example with the help of school staff [2;17;30;39]. Some studies were combinations of the ‘same for all’ and individual approaches. For example, these studies provided everyone with the same photographs but supplemented them with additional drawings, writings or the use of Makaton when needed [6,18] or prepared various modes of communication beforehand from which the participants chose individually [25].

Rephrasing complex concepts [19;25], other wordings in questions [1;2;6;7;10–13;24;39] or explaining the task again [31] were planned before or during data collection. The adaptation was reported in all instruments but observation (Table 2). In an individual interview, questions had alternative versions that were used if necessary [6]. In questionnaires, original Likert scales were rephrased into a ‘yes/no’ response format [10] or simpler scales were used [1;12;13, also 4], the names of friends and family were attached instead of ‘anyone’ [10], and affirmative responses were reduced by ending ‘yes/no’ questions into ‘or not’ [1]. In task‐based approaches, questions were built on initial student responses to get more detailed responses [18], and sentence starters were used [2].

The sub‐category ‘emotional support’ illustrated the encouragement of children in qualitative and quantitative studies. The adaptation was used with all instruments except observation (Table 2). The support was typically illustrated with the term ‘encouraging’: ‘[…] students were encouraged to consider their free‐time experiences. Students were encouraged to identify their favourite free‐time activities [17]. The adaptation included previously, or on‐the‐spot decided vocal [2;7;10–13;39] and physical (e.g., pointing) [19;35] prompts.

#### 3.2.2 Adaptations for Children With Moderate Intellectual Disabilities Who Communicate With Speech

Studies using the adaptations ‘experiences before testing’, ‘plain language’, ‘practising’, ‘reading aloud’ and ‘support people (family/friends or professionals)’ typically included children with moderate intellectual disabilities who communicated with speech. The sub‐category ‘experiences before testing’ shaped the data collection structure of quantitative studies with individual interviews and questionnaires (Table 2). Experiences were studied before ability testing with right and wrong answers, which would have been contradictory to the spirit of openness [10;27;35;40]. Plain language was reported in qualitative individual interviews [6;32;36] and a quantitative questionnaire [1] (Table 2). Interview guides were translated into easy‐to‐read material [32], and superfluous visual information [36], jargon and double‐barrel questions [6] were removed. Practising was used mainly in quantitative interviews and task‐based approaches where the participants practised how to label pictures before the actual tangible decision‐making sessions [15;35;40]. Reading aloud instructions and questions was a simple way to make questionnaires more accessible [10–13;26;29;31].

Professionals (e.g., school staff), family members and friends supported children with moderate intellectual disabilities and more severe disabilities in qualitative [2;5;6;17–19;36;41] and quantitative [1;30;35;38] studies. Support people were reported in all instruments but observation (Table 2). They supported participants by assisting them in communication [6], understanding and responding to questions [5;29;36;38;41] in addition to simply being present [2;29;35;36]. The role of support people was described as contradictory, especially in studies with children with severe ID, e.g., being in a central position in actualising the research while risking influencing the participants [2;17]. For studies that included only children with moderate intellectual disabilities who communicated with speech, it was reported that it was possible to minimise the supportive role and thus avoid contaminating influence [19;36].

#### 3.2.3 Adaptations for Children With Severe or Profound Intellectual Disabilities and (Non‐)symbolic Communication

Studies using the adaptations ‘consultation (family/friends or professionals)’, data collector (professionals), ‘functionality’, ‘non‐verbal cues’, ‘objects’, and ‘repetition’ typically included children with severe or profound intellectual disabilities and (non‐)symbolic communication. Parents interpreted their child's communication and affirmed the child's answers in uncertain situations to the researcher in a task‐based approach [25] (Table 2). In observation studies [21–23], parents played a major consultative role before and during data collection. Discussions were held about how the observations would occur, if the families had preferences about where they wanted the researcher to be placed physically, and how the parents could state their views during observations [21–23]. Researchers consulted professionals (school staff, organisation representatives) mainly before the data collection of qualitative individual interviews [35;39] and task‐based approaches [2;17;18;25]. The consultation included how to support communication and pose questions [39], and how to design the tasks [2;17;18].

Professionals facilitated tasks or conducted individual interviews for children with severe or profound intellectual disabilities who communicated without speech in three studies [18;30;39]. These professionals were familiar staff members who knew how to support the participants and adapt the data collection.

Functionality was an inseparable and initial part of some qualitative task‐based approach studies that included children with severe intellectual disabilities and (non‐)symbolic communication [2;17;18;25]. Functionality illustrated comprehensive or small details of playful, participative or otherwise functional elements. A range of tools with visual, audio, and tactile elements was created, from which the participants could choose. For example, children took photos and led the researchers around the community to show them things that the children found to be important [25]. Playful elements included drama [17;18], humour [18] and telling one's story with the help of a doll [25].

Non‐verbal cues (Huuhtanen 2012) were reported in six qualitative studies, which included children with severe or profound intellectual disabilities and (non‐)symbolic communication modes [5;17;18;21;25;39] and all instruments except questionnaires (Table 2). The studies acknowledged participants' facial expressions, body language and physical gestures, alongside their other communication. In interviews involving two interviewers, the other made notes of the participants' non‐verbal cues, getting a more robust representation of the experience [5, also 19]. Video recordings were useful when participants' communication skills ranged from speech to movement [17].

Objects of reference (Huuhtanen 2012) were used to support communication for children with severe or profound intellectual disabilities and (non‐)symbolic communication modes in individual interviews [30;39] and task‐based approaches [18;25]. In a task‐based approach, the participants explored a bag of objects that represented various life areas and interests [25]. A less usual example was executed when the researchers used props such as CDs and microphones to animate a session during which ‘news reporters’ asked participants the questions [18].

Repetition was used in qualitative task‐based approaches [17;18] and qualitative and quantitative individual interviews [5;35;36]. Four of the five studies included children with severe or profound ID, while all five included children who communicated without speech. Repetition included interviewing participants twice for clarification [4] and asking participants to check their earlier answers [35;36]. In a task‐based approach, a reminder of participants' earlier answers was presented to the group [18, also 17]. In addition, an earlier task was repeated due to a gap between sessions, and single questions were repeated from previous tasks for supportive information [18].

## 4 Discussion

This systematic review aimed to examine how the experiences of children with moderate to profound intellectual disabilities have been studied. Children with moderate intellectual disabilities who communicated with speech participated mainly in individual interviews and questionnaires. The popularity of individual interviews was not surprising considering its long traditions in qualitative research (Lune and Berg 2017, 65–93) and child‐centred research (Broström 2012) in addition to the support opportunities that the one‐on‐one setting provides. Quantitative questionnaires that rely on self‐reporting by children with (mild and) moderate intellectual disabilities seem to be a growing research field; it seems that questionnaires are increasingly adapted and validated specifically for children with intellectual disabilities (Maïano et al. 2023).

While observing studies that included children with severe and profound intellectual disabilities, the use of different terminology between countries was highlighted, and the categorisation into severe and profound intellectual disabilities became complex. Thus, it was vital to acknowledge the participants' modes of communication, such as relying on movements and gestures, although they were described as having severe disabilities. Within the group of children with severe or profound intellectual disabilities who communicated without speech, three research designs were highlighted: (1) individual interviews with multiple adaptations, (2) task‐based approaches in which functionality and active engagement were at the core of the instrument and (3) profound and long‐lasting observations. We do not imply that individual interviews suit everyone, but we argue that with the right adaptations, they suit *many* who are often excluded (e.g., symbolic communicators; children who are able to make decisions between two options).

However, task‐based approaches and profound and long‐lasting observations can provide participation opportunities for a wider group, such as children with profound intellectual and multiple disabilities and non‐symbolic communication (also Eisen et al. 2018; Gjermestad et al. 2022). Task‐based approaches, which do not have the same long traditions as the other four instruments, have become quite popular with participants with intensive support needs (Eisen et al. 2018; Gonzalez et al. 2020; Tesfaye et al. 2019). Some task‐based approaches found in the current review were developed particularly for the participants' varying abilities and could thus answer their needs more comprehensively. These studies reflected a proactive, inclusive design and embedded the adaptations into their designs. These designs included active engagement and multiple flexible, functional, playful and sensory elements. There should be an emphasis on the word *flexible*; the participation of children with non‐symbolic communication or profound intellectual and multiple disabilities can end up being quite passive in many activities, but by recognising their facial expressions, general body language and physical gestures, they too can communicate at many moments. To recognise these subtle signals, an adaptation of collaboration with parents or familiar professionals, either as a consultant or a data collector, was usually necessary (see Kruithof et al. 2024; Nieuwenhuijse et al. 2024). The profound and long‐lasting observation was reminiscent of ethnography and provided examples which managed to go somewhat deeper than simply observing separate actions (see also Mietola et al. 2017; Miettinen 2021; Skarsaune et al. 2021). These observations are highly interpretative, and the observer's communication skills are at the centre, but can provide opportunities to observe the small blinks of pre‐intentional expressions of the participants that are often not found with other methods (also Miettinen 2021; Skarsaune et al. 2021).

The studies usually used one of the five main data collection instruments to study children's experiences. Considering the methodological challenges, especially when studying the experiences of children with severe or profound intellectual disabilities who communicate non‐symbolically (Maes et al. 2020), we saw triangulating their data collection instruments as a focal way of producing trustworthy results and as a research gap in the field. However, research of this type is laborious. If the demands are too high, the research might not be completed. One or more proxies can provide valuable insights, either to be combined with the data collected directly from the child (Peltomäki et al. 2024) or as their own (Kruithof et al. 2024; Nieuwenhuijse et al. 2024). It is important that the data of the research field include both. Furthermore, a single task‐based approach can be a way of triangulating data collection methods if the approach produces data in several ways (e.g., photography and playful tasks).

The current review identified several adaptations. The adaptations designed for children with moderate intellectual disabilities who communicated with speech seemed quite easy to implement, while those used with children with severe or profound intellectual disabilities and (non‐)symbolic communication modes required more planning, resources, sensitivity and time. Simple adaptations in guidance were enough for many children with moderate intellectual disabilities. Collaboration was important in both groups but seemed indispensable and more comprehensive with children with severe and profound intellectual disabilities (see also Bailey et al. 2014; Gonzalez et al. 2020; Skarsaune et al. 2021; Tesfaye et al. 2019). As part of the collaboration, consulting the child's family and school professionals helped to design other adaptations. Similar consultation is common in school and care work and could be applied in research more often (see also Skarsaune et al. 2021). The consultation of family members and (familiar) professionals included them to interpret the child's answers to the researcher during data collection. We see this as a way of integrating the usefulness of proxy reports (Kruithof et al. 2024; Nieuwenhuijse et al. 2024) to research where the child is the participant. In addition to communication support that has been accentuated in earlier reviews (Bailey et al. 2014; Gonzalez et al. 2020; Tesfaye et al. 2019), our results highlight that several other adaptations can also be included.

When discussing adaptations, the trustworthiness of such research should be considered. While some studies reported the adaptations in detail and discussed their advantages and limitations, many studies reported the adaptations more generally, and these remain hard to perceive and reproduce (Bailey et al. 2014; Gonzalez et al. 2020; Tesfaye et al. 2019). Additionally, descriptions of encouragement were sometimes difficult to identify as adaptations of emotional support, as it is common in unstandardised interviews to encourage participants to lead the conversation (Lune and Berg 2017, 67–70). However, studies that reported emotional support in our review had data collected with quantitative questionnaires and structured interviews, in which encouragement is not usual. Encouraging was necessary to get (longer) answers, but also usually reported in brief, which might challenge evaluating the trustworthiness of the study. When doing research with children whose communication is fragile and acquiescence is usual, we thought that the aim should be to collect data as profoundly as possible from everyone. This means that individual solutions are necessary. However, this kind of research needs careful reporting, which can be done with separate appendices and method articles if the original article does not have space for all details.

Our MMAT evaluation showed that the quality of the studies varied (Appendix B). Eleven studies gained full scores (five ‘yes’ answers), and three studies gained fewer than three ‘yes’ answers. Ideally, we would have preferred to have more studies that reported adaptations amply with four or five ‘yes’ answers in the MMAT evaluation. Similar reviews have reported low (Bailey et al. 2014) or good (DePape and Lindsay 2015) overall methodological quality of studies, validating our findings.

### 4.1 Limitations

Our review has some limitations. Firstly, our categorisation of moderate, severe and profound intellectual disabilities was indicative only. This was an outcome of a variety of reporting and evaluation methods, in addition to the inaccuracy in some studies and differing terminologies between countries (Maes et al. 2020). We highlight that it is important to know which groups are being addressed, but concurrently, using the exact terms moderate, severe and profound intellectual disabilities is not always essential and ethical guidelines emphasise gathering and reporting only necessary personal data. These children have the right to participate in research without sharing every detail of their health, but without knowing some basic cognitive and communication skills, the methods and adaptations are difficult to reproduce.

Secondly, analysing whether an adaptation was made because of specific needs was sometimes ambiguous due to reporting issues. However, whether it was done because of specific needs was not essential; whether it was helpful was essential. In addition, the results of the intercoder reliability assessment were substantial or nearly perfect in all top‐level categories.

Finally, we might have missed relevant articles. This was difficult in our review, as the phenomena of interest were individual experiences of any topic, and all research designs and types were considered (Cooke et al. 2012). A more open search strategy would have risked making the screening too laborious and risking its trustworthiness, especially because the title and abstract screening was done by one researcher only. Future reviews should include non‐peer‐reviewed studies (e.g., doctoral dissertations) written in all languages.

## 5 Conclusions

This review highlights that individual solutions are often necessary when the experiences of children with moderate to profound intellectual disabilities are studied. We recommend that researchers report on the data collection process, its adaptations, and the participants' communication skills amply, and we hope that our presentation of various adaptations will assist researchers in planning, conducting and reporting such research. Furthermore, we urge researchers to continue to develop and triangulate data collection instruments and adaptations. Individual interviews with multiple well‐prepared adaptations, especially about communication support and collaboration with home and school, can include a variety of children. Task‐based approaches and in‐depth and profound observations can include children with severe and profound intellectual disabilities who communicate non‐symbolically and bring out new, unique and delicate perspectives.

## Conflicts of Interest

The authors declare no conflicts of interest.

## Supporting information


**Appendix S1** Search strategies of the systematic search.

**Appendix S2** Methodological quality appraisal of the eligible studies.

**Appendix S3** Descriptions of all adaptations.

**Appendix S4** Reference list of the eligible studies.

## Data Availability

Once the article is published, the template data collection forms and analytic code will be added to Zenodo under the CC BY Licence. Due to copyrights, data extracted from the included studies and data used for all analyses cannot be published. However, they can be requested from the corresponding author.
