# Supplementary material for: Studying the Experiences of Children With Moderate to Profound Intellectual Disabilities in Research: A Systematic Review
Source: J Intellect Disabil Res. 2025 Jul 12;70(1):1–15. doi: 10.1111/jir.70009 (PMC12703066; doi:10.1111/jir.70009)
Supplement: Supplementary file 1 — Appendix S1 Search strategies of the systematic search. [file JIR-70-1-s002.docx]

Appendix A. Search strategies of the systematic search.

Table A1. Education Collection, Social Science Database and Sociology Collection search strategy

| Databases: Education Collection, Social Science Database, Sociology Collection (ProQuest)  Date: 29/01/2025 | | |
| --- | --- | --- |
| Set# | Search term(s) | Number of hits |
| 1 | summary(intellect* OR developmental OR learning) OR title(intellect* OR developmental OR learning) | 1,092,607 |
| 2 | summary(disab*) OR title(disab*) | 127,586 |
| 3 | summary(moderate* OR severe* OR profound*) OR title(moderate* OR severe* OR profound*) | 275,737 |
| 4 | summary(experience* OR view OR views OR participatory* OR inclusive* OR own voice* OR authentic voice* OR giving voice*) OR title(experience* OR view OR views OR participatory* OR inclusive* OR own voice* OR authentic voice* OR giving voice*) | 1,490,003 |
| 5 | summary(child* OR adolescen* OR kid OR kids OR youth* OR teen OR teens OR teenage*) OR title(child* OR adolescen* OR kid OR kids OR youth* OR teen OR teens OR teenage*) | 1,529,823 |
| 6 | (summary(child* OR adolescen* OR kid OR kids OR youth* OR teen OR teens OR teenage*) OR title(child* OR adolescen* OR kid OR kids OR youth* OR teen OR teens OR teenage*)) AND (summary(experience* OR view OR views OR participatory* OR inclusive* OR own voice* OR authentic voice* OR giving voice*) OR title(experience* OR view OR views OR participatory* OR inclusive* OR own voice* OR authentic voice* OR giving voice*)) AND (summary(moderate* OR severe* OR profound*) OR title(moderate* OR severe* OR profound*)) AND (summary(disab*) OR title(disab*)) AND (summary(intellect* OR developmental OR learning) OR title(intellect* OR developmental OR learning)) | 464 |
| 7 | (summary(child* OR adolescen* OR kid OR kids OR youth* OR teen OR teens OR teenage*) OR title(child* OR adolescen* OR kid OR kids OR youth* OR teen OR teens OR teenage*)) AND (summary(experience* OR view OR views OR participatory* OR inclusive* OR own voice* OR authentic voice* OR giving voice*) OR title(experience* OR view OR views OR participatory* OR inclusive* OR own voice* OR authentic voice* OR giving voice*)) AND (summary(moderate* OR severe* OR profound*) OR title(moderate* OR severe* OR profound*)) AND (summary(disab*) OR title(disab*)) AND (summary(intellect* OR developmental OR learning) OR title(intellect* OR developmental OR learning)) AND (la.exact("ENG") AND pd(20000101-20250129) AND PEER(yes)) | 326 |

Table A2. CINAHL search strategy

| Database: CINAHL (EBSCO)  Date: 29/01/2025 | | | |
| --- | --- | --- | --- |
| Set# | Search term(s) | Limiters | Number of hits |
| 1 | ( intellect* OR developmental OR learning ) AND ( disab* ) AND ( moderate* OR severe* OR profound* ) |  | 6,749 |
| 2 | ( experience* OR view OR views OR participatory* OR inclusive* OR own voice* OR authentic voice* OR giving voice* ) |  | 726,849 |
| 3 | S1 AND S2 |  | 1,239 |
| 4 | S1 AND S2 | Published Date: 20000101-20250129; Peer Reviewed; Language: English, Finnish | 1095 |
| 5 | S1 AND S2 | Published Date: 20000101-20250129; Peer Reviewed; Age Groups: All infant, All Child; Language: English, Finnish | 472 |

Table A3. APA PsychInfo search strategy

| Databases: APA PsychInfo (Ovid) | | Date, Number of hits | |
| --- | --- | --- | --- |
| Set# | Search term(s) | 07/11/2023 | 2024–29/01/2025 |
| 1 | (intellect* or developmental or learning).mp. | 843,084 | 27,430 |
| 2 | disab*.mp. | 205,316 | 6,514 |
| 3 | 1 and 2 | 96,701 | 2,752 |
| 4 | (moderate* or severe* or profound*).mp. | 347,456 | 16,585 |
| 5 | (experience* or view or views or participatory* or inclusive* or own voice* or authentic voice* or giving voice*).mp. | 1,045,254 | 50,301 |
| 6 | 3 and 4 and 5 | 2,349 | 136 |
| 7 | limit 6 to peer reviewed journal | 1,719 | 95 |
| 8 | limit 7 to (childhood or adolescence <13 to 17 years>) | 706 | 22 |
| 9 | limit 8 to ((english or finnish) and yr="2000 -Current") | 490 | 22 |
