# Supplementary material for: Studying the Experiences of Children With Moderate to Profound Intellectual Disabilities in Research: A Systematic Review
Source: J Intellect Disabil Res. 2025 Jul 12;70(1):1–15. doi: 10.1111/jir.70009 (PMC12703066; doi:10.1111/jir.70009)
Supplement: Supplementary file 3 — Appendix S3 Descriptions of all adaptations. [file JIR-70-1-s001.docx]

Appendix C. Descriptions of all adaptations.

| Top-level category | Sub-category | Description |
| --- | --- | --- |
| Methodological principles | Experiences before testing | Data of experiences are collected before any kind of ability testing, which includes right and wrong answers. |
|  | From knowing to knowledge | The aim was to get to know the participants before data collection.  This can include a separate meeting session or an activity that aims to get to know each other before data collection starts.  The aim is to build a trusting relationship.  By knowing the participants, it is reasoned to get the data collected better and to get better data.  The data collection can be planned based on participants. |
|  | From one instrument to another | A questionnaire is turned into an individual interview for the target group to participate. |
|  | Functionality | The data collection can be designed altogether functional.  It can also only partly be functional.  The data are collected with these playful/participative/functional activities. |
|  | Time is flexible | The duration of data collection (e.g., how long an interview session lasts) depends on the participant’s individual needs.  Breaks are given as necessary, and sessions can be divided into different days. |
| Communication support | Auditive | Music and sound (other than speech) are used to support communication. |
|  | Drawing/writing | Questions can be answered by drawing and writing in addition to speaking. |
|  | Non-verbal cues | Non-verbal answers are acknowledged alongside other answers.  Answers told by movement and gestures are listened to.  Communication is seen as completeness, and body language, gestures and other non-verbal cues are part of it. |
|  | Objects | Different objects are used to support communication.  They can be touched.  Objects can be used to facilitate additional answers. |
|  | Pictures/symbols /signing | Communication is supported with pictures (e.g., Picture Communication Symbols®, photographs), different symbols and key word signing.  Pictures and symbols can be presented in large print, so it is easier to see and handle them.  They can also be made otherwise clearer, e.g., by modifying dimensions. |
|  | Plain language | The researcher alters the questions and explanations to a simplified language. |
| Guidance | Asking examples | The participant is asked to provide an example to support their answer.  Asking for examples can be used to validate the correctness of an answer. |
|  | Cutting-off questions | Questions of an original questionnaire are cut off, so there would not be too many questions, and the participants have the energy to answer them. |
|  | Emotional support | Participants are described as needing to be encouraged to answer questions.  Encouragement is rarely described in detail.  Emotional support is delivered by speech.  It can also be a physical prompt with an object, for example. |
|  | Peer support | Participants stimulate or support each other.  The interaction between participants can create answers or supplement them.  Participants can discuss with each other and share selections. |
|  | Practising | The participant practices answering before data collection.  Includes usually a tool/device, that is good to be familiar with before the data collection starts. |
|  | Providing examples | The researcher(s) provides the participant with an example to clarify the issue.  The example can be a presentation of two opposite answers and thus reduce acquiescence. |
|  | Reading aloud | The researcher reads the questions/instructions aloud to the participants, in addition to having them in a written form. |
|  | Repetition | A previous activity is repeated.  A question is asked again later.  Participants are told what they have previously answered.  Participants are asked to check their responses. |
|  | Rephrasing | Question/word/sentence is rephrased.  This can be done before or during data collection.  The choice can be simplified by reducing their amount.  A difficult concept can be altered.  An abstract word can be rephrased with a more concrete option.  An unknown word can be changed to a more familiar or meaningful for the participant.  A rephrasing can be planned and used if necessary.  A long sentence/instruction/other can be divided into multiple parts. |
| Collaboration | Consultation (co-researcher) | Children are consulted before or during data collection.  The tasks or questions are designed with guidance of the children. |
|  | Consultation (family/friends) | Family/friends (usually parents) are consulted before or during data collection.  They can assist with how to support communication or what kinds of questions to ask.  They can interpret the participant’s answers to the researcher during data collection.  They can confirm answers.  As opposed to being a support person, here, the interaction is between the researcher and the family member/friend. |
|  | Consultation (professionals) | Professionals (e.g., teachers or an organisation representative) are consulted before or during data collection.  They can assist with how to support communication or what kinds of questions to ask.  They can interpret the child’s answers to the researcher during data collection.  They can confirm answers.  As opposed to being a support person, here, the interaction is between the researcher and the professional. |
|  | Data collector (co-researcher) | A co-researcher collects the data among others or alone. |
|  | Data collector (professionals) | Another professional, typically a staff member, collects the data instead of the researchers.  This is often explained by them knowing the participants and how they communicate. |
|  | Support people (family/friends) | As opposed to being in a consultative role, here, the interaction is between a family member/friend (usually parent) and the participant.  The support person supports the participant as needed, usually with communication.  Their role can be solely being present. |
|  | Support people (professionals) | As opposed to being in a consultative role, here, the interaction is between the professional and the participant.  The support person supports the participant as needed, usually with communication. |
