# Supplementary material for: Studying the Experiences of Children With Moderate to Profound Intellectual Disabilities in Research: A Systematic Review
Source: J Intellect Disabil Res. 2025 Jul 12;70(1):1–15. doi: 10.1111/jir.70009 (PMC12703066; doi:10.1111/jir.70009)
Supplement: Supplementary file 4 — Appendix S4 Reference list of the eligible studies. [file JIR-70-1-s003.docx]

Appendix D. Reference list of the eligible studies.

| No. | Reference |
| --- | --- |
| 1 | Aderemi, T. J. & Pillay, B. J. (2013) Sexual abstinence and HIV knowledge in school-going adolescents with intellectual disabilities and non-disabled adolescents in Nigeria. *Journal of Child & Adolescent Mental Health* **25**, 161–74. http://doi.org/10.2989/17280583.2013.823867 |
| 2 | Allen, G., Milne, B., Velija, P. & Radley, R. (2022) ‘Hearing their voice’: The experiences of physical education with pupils diagnosed with severe learning disabilities. *Sport, Education and Society* **29**, 342–57. https://doi.org/10.1080/13573322.2022.2141704 |
| 3 | Aviram, R., Khvorostianov, N., Harries, N. & Bar-Haim, S. (2021) Perceived barriers and facilitators for increasing the physical activity of adolescents and young adults with cerebral palsy: A focus group study. *Disability and Rehabilitation* **44**, 6649–59. https://doi.org/10.1080/09638288.2021.1970252 |
| 4 | Benini, F., Trapanotto, M., Gobber, D., Agosto, C., Carli, G., Drigo, P. et al. (2004) Evaluating pain induced by venipuncture in pediatric patients with developmental delay. *The Clinical Journal of Pain* **20**, 156–63. https://doi.org/10.1097/00002508-200405000-00005 |
| 5 | Bennett, S., Gallagher, T., Shuttleworth, M., Somma, M. & White, R. (2017) Teen dreams: Voices of students with intellectual disabilities. *Journal on Developmental Disabilities* **23**, 64–75. |
| 6 | Boyden, P., Muniz, M. & Laxton-Kane, M. (2012) Listening to the views of children with learning disabilities: An evaluation of a learning disability CAMHS service. *Journal of Intellectual Disabilities* **17**, 51–63. <http://doi.org/10.1177/1744629512469923> |
| 7 | Byrne, A. & Hennessy, E. (2009) Understanding challenging behaviour: Perspectives of children and adolescents with a moderate intellectual disability. *Journal of Applied Research in Intellectual Disabilities* **22**, 317–25. https://doi.org/10.1111/j.1468-3148.2008.00465.x |
| 8 | Caton, S. & Kagan, C. (2007) Comparing transition expectations of young people with moderate learning disabilities with other vulnerable youth and with their non‐disabled counterparts. *Disability & Society* **22**, 473–88. <https://doi.org/10.1080/09687590701427586> |
| 9 | Connaughton, H. & Cline, T. (2020) How far can social role valorisation theory help in transition planning for a school‐leaver with significant special needs? *British Journal of Learning Disabilities* **49**, 80–6. https://doi.org/10.1111/bld.12310 |
| 10 | Cooney, G., Jahoda, A., Gumley, A. & Knott, F. (2006) Young people with intellectual disabilities attending mainstream and segregated schooling: Perceived stigma, social comparison and future aspirations. *Journal of Intellectual Disability Research* **50**, 432–44. https://doi.org/10.1111/j.1365-2788.2006.00789.x |
| 11 | Dubé, C., Morin, A. J., Olivier, E., Gilbert, W., Tracey, D., Craven, R. G. & Maïano, C. (2023) School experiences and anxiety trajectories among youth with intellectual disabilities. *Journal of Autism and Developmental Disorders* **54**, 4111–29. https://doi.org/10.1007/s10803-023-06127-y |
| 12 | Dubé, C., Morin, A. J., Olivier, E., Tóth-Király, I., Tracey, D., Craven, R. G. & Maïano, C. (2022a) Longitudinal associations between relationship quality and depression among youth with intellectual disabilities: A latent change perspective. *Journal of Autism and Developmental Disorders* **54**, 673–90. https://doi.org/10.1007/s10803-022-05805-7 |
| 13 | Dubé, C., Morin, A. J., Tóth-Király, I., Olivier, E., Tracey, D., McCune, V. S., Craven, R. G. & Maïano, C. (2022b) Social interaction profiles among youth with intellectual disabilities: Associations with indicators of psychosocial adjustment. *Journal of Autism and Developmental Disorders* **54**, 458–76. https://doi.org/10.1007/s10803-022-05783-w |
| 14 | Fairhurst, C., Shortland, A., Chandler, S., Will, E., Scrutton, D., Simonoff, E. et al. (2018) Factors associated with pain in adolescents with bilateral cerebral palsy. *Developmental Medicine & Child Neurology* **61**, 929–36. <https://doi.org/10.1111/dmcn.14113> |
| 15 | Ferreira, M., Aguiar, C., Correia, N., Fialho, M. & Pimentel, J. S. (2016) Social experiences of children with disabilities in inclusive Portuguese preschool settings. *Journal of Early Intervention* **39**, 33–50. https://doi.org/10.1177/1053815116679414 |
| 16 | Ferreira, M., Aguiar, C., Correia, N., Fialho, M. & Pimentel, J. S. (2019) Friendships and social acceptance of Portuguese children with disabilities: The role of classroom quality, individual skills, and dosage. *Topics in Early Childhood Special Education* **39**, 183–95. https://doi.org/10.1177/0271121419864419 |
| 17 | Fitzgerald, H. (2007) Dramatizing physical education: using drama in research. *British Journal of Learning Disabilities* **35**, 253–60. https://doi.org/10.1111/j.1468-3156.2007.00471.x |
| 18 | Fitzgerald, H., Jobling, A. & Kirk, D. (2003) Listening to the ‘voices’ of students with severe learning difficulties through a task‐based approach to research and learning in physical education. *Support for Learning* **18**, 123–9. https://doi.org/10.1111/1467-9604.00294 |
| 19 | Foley, K. R., Blackmore, A. M., Girdler, S., O’Donnell, M., Glauert, R., Llewellyn, G. et al. (2012) To feel belonged: The voices of children and youth with disabilities on the meaning of wellbeing. *Child Indicators Research* **5**, 375–91. https://doi.org/10.1007/s12187-011-9134-2 |
| 20 | Hatton, C., Emerson, E., Robertson, J. & Baines, S. (2017) The mental health of adolescents with and without mild/moderate intellectual disabilities in England: Secondary analysis of a longitudinal cohort study. *Journal of Applied Research in Intellectual Disabilities* **31**, 768–77. https://doi.org/10.1111/jar.12428 |
| 21 | Hingley-Jones, H. (2009) Developing practice-near social work research to explore the emotional worlds of severely learning-disabled adolescents in ‘transition’ and their families. *Journal of Social Work Practice* **23**, 413–28. https://doi.org/10.1080/02650530903374952 |
| 22 | Hingley-Jones, H. (2011) An exploration of the use of infant observation methods to research the identities of severely learning-disabled adolescents and to enhance relationship-based practice for professional social work. *Infant Observation* **14**, 317–33. https://doi.org/10.1080/13698036.2011.616305 |
| 23 | Hingley‐Jones, H. (2012) Emotion and relatedness as aspects of the identities of adolescents with severe learning disabilities: Contributions from ‘practice‐near’social work research. *Child & Family Social Work* **18**, 458–66. https://doi.org/10.1111/j.1365-2206.2012.00865.x |
| 24 | Hughes, C., Cosgriff, J. C., Agran, M. & Washington, B. H. (2013) Student self-determination: A preliminary investigation of the role of participation in inclusive settings. *Education and Training in Autism and Developmental Disabilities* **48**, 3–17. |
| 25 | Jenkin, E., Wilson, E., Clarke, M., Campain, R. & Murfitt, K. (2017) Listening to the voices of children: understanding the human rights priorities of children with disability in Vanuatu and Papua New Guinea. *Disability & Society* **32**, 358–80. https://doi.org/10.1080/09687599.2017.1296348 |
| 26 | Kumas, O. A. & Yıldırım, A. E. S. (2024) Development of early numeracy skills in children with moderate intellectual disability. *Journal of Intellectual Disabilities* **28**, 343–58. <https://doi.org/10.1177/17446295241228897> |
| 27 | Larkin, P., Jahoda, A., Macmahon, K. & Pert, C. (2011) Interpersonal sources of conflict in young people with and without mild to moderate intellectual disabilities at transition from adolescence to adulthood. *Journal of Applied Research in Intellectual Disabilities* **25**, 29–38. https://doi.org/10.1111/j.1468-3148.2011.00652.x |
| 28 | Medved, M. I. & Brockmeier, J. (2004) Making sense of traumatic experiences: Telling your life with Fragile X syndrome. *Qualitative Health Research* **14**, 741–59. <https://doi.org/10.1177/1049732304265972> |
| 29 | Noerr, K. L. & Swinford, R. (2024) The influence of assistance in home‐based exercise programmes for individuals with intellectual disabilities. *Journal of Intellectual Disability Research* **69**, 55–64. <https://doi.org/10.1111/jir.13191> |
| 30 | Pearlman, S. & Michaels, D. (2019) Hearing the voice of children and young people with a learning disability during the Educational Health Care Plan (EHCP). *Support for Learning* **34**, 148–61. https://doi.org/10.1111/1467-9604.12245 |
| 31 | Pijl, S. J. & Frostad, P. (2010) Peer acceptance and self‐concept of students with disabilities in regular education. *European Journal of Special Needs Education* **25**, 93–105. https://doi.org/10.1080/08856250903450947 |
| 32 | Retznik, L., Wienholz, S., Höltermann, A., Conrad, I. & Riedel-Heller, S. G. (2021) “It tingled as if we had gone through an anthill.” Young people with intellectual disability and their experiences with relationship, sexuality and contraception. *Sexuality and Disability* **39**, 421–38. https://doi.org/10.1007/s11195-020-09670-z |
| 33 | Ring, E. & Travers, J. (2005) Barriers to inclusion: A case study of a pupil with severe learning difficulties in Ireland. *European Journal of Special Needs Education* **20**, 41–56. https://doi.org/10.1080/0885625042000319070 |
| 34 | Robertson, J., Emerson, E., Baines, S. & Hatton, C. (2018) Self-reported participation in sport/exercise among adolescents and young adults with and without mild to moderate intellectual disability. *Journal of Physical Activity and Health* **15**, 247–54. <https://doi.org/10.1123/jpah.2017-0035> |
| 35 | Samuelsson, J., Holmer, E., Johnels, J. Å., Palmqvist, L., Heimann, M., Reichenberg, M. & Thunberg, G. (2023) My point of view: Students with intellectual and communicative disabilities express their views on speech and reading using Talking Mats. *British Journal of Learning Disabilities* **52**, 23–35. https://doi.org/10.1111/bld.12543 |
| 36 | Valiquette, C., Sutton, A. & Ska, B. (2010) A graphic symbol tool for the evaluation of communication, satisfaction and priorities of individuals with intellectual disability who use a speech generating device. *Child Language Teaching and Therapy* **26**, 303–19. https://doi.org/10.1177/0265659010369281 |
| 37 | Vogel, G. & Reiter, S. (2003) Spiritual dimensions of bar/bat mitzvah ceremonies for Jewish children with developmental disabilities. *Education and Training in Developmental Disabilities* **38**, 314–22. |
| 38 | Warnick, J. E., Pillay, J. & Munongi, L. (2024) Risk factors related to the mental health issues of selected South African adolescent learners living with mild to moderate intellectual difficulties. *Advances in Mental Health and Intellectual Disabilities* **18**, 110–24. https://doi.org/10.1108/AMHID-02-2024-0009 |
| 39 | Whitehurst, T. (2006) Liberating silent voices–perspectives of children with profound & complex learning needs on inclusion. *British Journal of Learning Disabilities* **35**, 55–61. <https://doi.org/10.1111/j.1468-3156.2006.00405.x> |
| 40 | Young, R., Dagnan, D. & Jahoda, A. (2015) Leaving school: A comparison of the worries held by adolescents with and without intellectual disabilities. *Journal of Intellectual Disability Research* **60**, 9–21. https://doi.org/10.1111/jir.12223 |
| 41 | Young-Southward, G., Cooper, S. A. & Philo, C. (2017) Health and wellbeing during transition to adulthood for young people with intellectual disabilities: A qualitative study. *Research in Developmental Disabilities* **70**, 94–103. <https://doi.org/10.1016/j.ridd.2017.09.003> |
